# Supplementary material for: Direct estimation of genotype fitness from time series
Source: bioRxiv. 2026 Jul 20:2026.07.18.739367. Preprint. [Version 1] doi: 10.64898/2026.07.18.739367 (PMC13419391; doi:10.64898/2026.07.18.739367)
Supplement: Supplement 1 [file NIHPP2026.07.18.739367v1-supplement-1.pdf]

bioRxiv preprint doi: https://doi.org/10.64898/2026.07.18.739367; this version posted July 20, 2026. The copyright holder for this preprint (which was not certified by peer review) is the author/funder, who has granted bioRxiv a license to display the preprint in perpetuity. It is made available under a CC-BY 4.0 International license.

Supplementary Information for “Direct estimation of genotype fitness from time series”

Vaibhav Mohanty[1,2,3,*] **and Eugene I. Shakhnovich**[1,*]

[1] *Department of Chemistry and Chemical Biology, Harvard University, Cambridge, MA 02138*

[2] *Harvard/MIT MD-PhD Program, Harvard Medical School, Boston, MA 02115 and Massachusetts Institute of Technology, Cambridge, MA 02139*

[3] *Program in Health Sciences and Technology, Harvard Medical School, Boston, MA 02115 and Massachusetts Institute of Technology, Cambridge, MA 02139*

Contents

S1 Fitness can be estimated from genotype correlation time series data 2

S2 Alternate equation for exactly known mutation rate matrix 3

S3 Alternate equation in the strong selection, weak mutation limit 3

S3.1 Zero Mutation Matrix 4

S3.2 Proof of exactness of Equation (S3.3) for a sequence of selective sweeps 4

S4 Derivation of negative identity matrix approximation for scaled mutation matrix for large genotype spaces 8

S5 Supplementary Figures 12

[*] Correspondence: mohanty@hms.harvard.edu (V.M.) and shakhnovich@chemistry.harvard.edu (E.I.S.)

bioRxiv preprint doi: https://doi.org/10.64898/2026.07.18.739367; this version posted July 20, 2026. The copyright holder for this preprint (which was not certified by peer review) is the author/funder, who has granted bioRxiv a license to display the preprint in perpetuity. It is made available under a CC-BY 4.0 International license.

S1 Fitness can be estimated from genotype correlation time series data

Consider a population of N haploid, asexually reproducing organisms. The forward-time, continuous-time evolution of frequency f_g of a genotype g is given by the stochastic differential equation:

$$\frac{\partial f_g}{\partial t} = \underbrace{f_g\left(X_g - \sum_{g'} X_{g'} f_{g'}\right)}_{\text{selection}} + \underbrace{\sum_{g' \neq g}\left(\mu_{g' \to g} f_{g'} - \mu_{g \to g'} f_g\right)}_{\text{mutation}} + \underbrace{\sum_{g'}\left(\delta_{g,g'} - f_g\right)\sqrt{\frac{f_{g'}}{N}}\eta_{g'}(t)}_{\text{genetic drift}}, \tag{S1.1}$$

where X_g is the fitness of genotype g, $\mu_{g \to g'}$ is the mutation rate from genotype g to g', and $\eta_{g'}(t)$ is a Gaussian noise term with $\langle \eta_{g'}(t) \rangle = 0$ and $\langle \eta_g(t)\eta_{g'}(t') \rangle = \delta_{g,g'}\delta(t - t')$. In the limit of large population ($N \to \infty$), we can approximate the diffusion term to be small relative to the selection and mutation terms. These operations cause the above equation to now become

$$\frac{\partial \mathbf{f}}{\partial t} = [\mathrm{diag}(\mathbf{f}(t)) - \mathbf{f}(t) \otimes \mathbf{f}(t)]\mathbf{X} + M\mathbf{f}(t). \tag{S1.2}$$

We now perform time averaging to obtain

$$\frac{\mathbf{f}(T) - \mathbf{f}(0)}{T} = [\mathrm{diag}(\langle \mathbf{f}(t) \rangle_t) - \langle \mathbf{f}(t) \otimes \mathbf{f}(t) \rangle_t]\mathbf{X} + M \langle \mathbf{f}(t) \rangle_t \tag{S1.3}$$

where $\langle \cdot \rangle_t = \frac{1}{T}\int_0^T \mathrm{d}t \cdot$ denotes time averaging, and we have defined a mutation rate matrix $M_{gg'} = \mu_{g' \to g}$ for $g \neq g'$ and $M_{gg} = -\sum_{g' \neq g} \mu_{g \to g'}$. Recognizing that absolute mutation rates may be affected by time discretization, we use a rescaled relative mutation rate matrix $\widetilde{M} = \alpha M$, with α being a scaling factor. Similarly, only relative fitnesses affect the evolutionary dynamics, so the fitnesses $\mathbf{X}$ should absorb any scaling factor. Letting $\mathbf{X} = \beta\widetilde{\mathbf{X}}/\alpha$, choosing β/α as a scaling factor, and rearranging into a matrix equation, we have

$$-\widetilde{M}\langle \mathbf{f}(t) \rangle_t = \left[\mathrm{diag}(\langle \mathbf{f}(t) \rangle_t) - \langle \mathbf{f}(t) \otimes \mathbf{f}(t) \rangle_t \;\middle|\; \frac{\mathbf{f}(0) - \mathbf{f}(T)}{T}\right]\begin{bmatrix}\beta\widetilde{\mathbf{X}} \\ \alpha\end{bmatrix}, \tag{S1.4}$$

The estimated relative fitness landscape $\hat{\widetilde{\mathbf{X}}}$ can be obtained from linear regression by minimizing the least squares loss of the right and left sides of the equation, above, computable with the pseudoinverse

$$\begin{bmatrix}\beta\hat{\widetilde{\mathbf{X}}} \\ \hat{\alpha}\end{bmatrix} = -\left[\mathrm{diag}(\langle \mathbf{f}(t) \rangle_t) - \langle \mathbf{f}(t) \otimes \mathbf{f}(t) \rangle_t \;\middle|\; \frac{\mathbf{f}(0) - \mathbf{f}(T)}{T}\right]^{+}\widetilde{M}\langle \mathbf{f}(t) \rangle_t \tag{S1.5}$$

The Moore-Penrose pseudoinverse form $A^{+} = (A^T A)^{-1}A^T$ does not exist because $(A^T A)^{-1}$ is singular, since A has one more column than rows. But, the pseudoinverse always exists and can be calculated with singular value decomposition (SVD). If $A = U\Sigma V^T$ is the SVD of A, then $A^{+} = V\Sigma^{-1}U^T$, which is approximated by $A^{+} = V\widetilde{\Sigma}^{-1}U^T$, where $\widetilde{\Sigma}$ has the smallest singular values removed.

bioRxiv preprint doi: https://doi.org/10.64898/2026.07.18.739367; this version posted July 20, 2026. The copyright holder for this preprint (which was not certified by peer review) is the author/funder, who has granted bioRxiv a license to display the preprint in perpetuity. It is made available under a CC-BY 4.0 International license.

S2 Alternate equation for exactly known mutation rate matrix

In the infinite population limit $N \to \infty$, we have

$$\frac{\partial \mathbf{f}}{\partial t} = [\mathrm{diag}(\mathbf{f}(t)) - \mathbf{f}(t) \otimes \mathbf{f}(t)]\mathbf{X} + M\mathbf{f}(t). \tag{S2.1}$$

We now perform time averaging to obtain

$$\frac{\mathbf{f}(T) - \mathbf{f}(0)}{T} = [\mathrm{diag}(\langle \mathbf{f}(t) \rangle_t) - \langle \mathbf{f}(t) \otimes \mathbf{f}(t) \rangle_t]\mathbf{X} + M \langle \mathbf{f}(t) \rangle_t \tag{S2.2}$$

where $\langle \cdot \rangle_t = \frac{1}{T}\int_0^T \mathrm{d}t \cdot$ denotes time averaging, and we have defined a mutation rate matrix $M_{gg'} = \mu_{g' \to g}$ for $g \neq g'$ and $M_{gg} = -\sum_{g' \neq g} \mu_{g \to g'}$. When the mutation rate is exactly known (with respect to the correct time coarse-graining), a modified inference protocol can be used which does not involve rectangular matrices. We can rearrange the above equation to obtain We now perform time averaging to obtain

$$\hat{\mathbf{X}} = [\mathrm{diag}(\langle \mathbf{f}(t) \rangle_t) - \langle \mathbf{f}(t) \otimes \mathbf{f}(t) \rangle_t]^+ \left(\frac{\mathbf{f}(T) - \mathbf{f}(0)}{T} - M \langle \mathbf{f}(t) \rangle_t \right). \tag{S2.3}$$

The pseudoinverse becomes an inverse when the matrix is invertible.

S3 Alternate equation in the strong selection, weak mutation limit

In the strong selection, weak mutation (SSWM) limit, mutation rates are typically much slower than fitnesses (growth rates). As a result, the evolutionary dynamics are typically dominated by selective sweeps in which one genotype fixes in the population before the next mutation is introduced. This leads to principally monomorphic dynamics in which the entire population tends to be concentrated at one genotype, then another genotype, then another, and so on.

Like in the main text, we recognize that absolute mutation rates may be affected by time discretization, we use a rescaled relative mutation rate matrix $\alpha\widetilde{M} = M$, with α being a scaling factor (note that the convention is slightly different from the rescaling in the main text). We again rearrange eq. (S2.2) into a matrix equation, but including the mutation term in the matrix to be pseudoinverted. This leads to an equation that is the same as what we presented in the main text, but with the boundary term and the mutation term swapped:

$$\frac{\mathbf{f}(T) - \mathbf{f}(0)}{T} = \left[\mathrm{diag}(\langle \mathbf{f}(t) \rangle_t) - \langle \mathbf{f}(t) \otimes \mathbf{f}(t) \rangle_t \;\middle|\; \widetilde{M} \langle \mathbf{f}(t) \rangle_t \right] \begin{bmatrix} \mathbf{X} \\ \alpha \end{bmatrix}, \tag{S3.1}$$

The estimated relative fitness landscape $\hat{\tilde{\mathbf{X}}}$ can be obtained from linear regression by minimizing the least squares loss of the right and left sides of the equation, above, computable with the pseudoinverse

$$\begin{bmatrix} \hat{\mathbf{X}} \\ \hat{\alpha} \end{bmatrix} = \left[\mathrm{diag}(\langle \mathbf{f}(t) \rangle_t) - \langle \mathbf{f}(t) \otimes \mathbf{f}(t) \rangle_t \;\middle|\; \widetilde{M} \langle \mathbf{f}(t) \rangle_t \right]^+ \left(\frac{\mathbf{f}(T) - \mathbf{f}(0)}{T} \right) \tag{S3.2}$$

bioRxiv preprint doi: https://doi.org/10.64898/2026.07.18.739367; this version posted July 20, 2026. The copyright holder for this preprint (which was not certified by peer review) is the author/funder, who has granted bioRxiv a license to display the preprint in perpetuity. It is made available under a CC-BY 4.0 International license.

Once again, the exact pseudoinverse $A^+ = (A^T A)^{-1} A^T$ does not exist because $(A^T A)^{-1}$ is singular. If $A = U\Sigma V^T$ is the SVD of A, then $A^+ = V\Sigma^{-1}U^T$, which is approximated by $A^+ = V\widetilde{\Sigma}^{-1}U^T$, where $\widetilde{\Sigma}$ has the smallest singular values removed.

S3.1 Zero Mutation Matrix

When truly zero mutations are present, the mutation matrix vanishes, so only the derivative term $\frac{\partial \mathbf{f}}{\partial t}$ can contribute to the inference process. In this case, we simply use eq. (S3.3), but since $M = 0$, we can simply remove the extra column from the pseudoinverted matrix (and the α parameter is thus arbitrary):

$$\hat{\mathbf{X}} = [\mathrm{diag}(\langle \mathbf{f}(t)\rangle_t) - \langle \mathbf{f}(t) \otimes \mathbf{f}(t)\rangle_t]^+ \left(\frac{\mathbf{f}(T) - \mathbf{f}(0)}{T}\right) \tag{S3.3}$$

S3.2 Proof of exactness of Equation (S3.3) for a sequence of selective sweeps

We now analytically consider the case of n selective sweeps occurring in an infinite population hopping from genotype to genotype in a sequence of increasing fitness. We will prove that eq. (S3.3) yields fitness estimates that have Pearson correlation $r = 1$ with the original fitnesess.

Consider a set of genotypes labeled by indices $\{1, \ldots, n\}$ and their fitnesses $X_1 < X_2 < \cdots < X_n$. In our scenario, $f_1(0) = 1 - \epsilon$ (for $0 < \epsilon < 1/2$) fraction of the population begins at genotype 1 at time $t = 0$ while the remaining ϵ fraction of the population is on genotype 2, so $f_2(0) = \epsilon$. As a result, over a time window $T_{1\to2}$, genotype 2 will fix in the population and the fraction of population 1 will become small. In particular, we will define $T_{1\to2}$ to be so that at some point infinitesimally before time $t = T_{1\to2}$, fraction of genotype 1 becomes $f_1(T_{1\to2}) = \epsilon$ and $f_1(t \to T^-_{1\to2}) = 1 - \epsilon$. At this time, a very rare mutation occurs in which genotype 1 disappears (this can also follow from assuming that N is large but finite) and genotype 3 appears at frequency $f_3(t \to T^+_{1\to2}) = \epsilon$. Another selective sweep then occurs over a time $T_{2\to3}$ in which genotype 3 fixes in the population, sending the frequency of genotype 2 eventually to ϵ. This procedure repeats in a sequence of increasing fitness so that ultimately the time series ends with $f_n(T) = 1-\epsilon$ and $f_{n-1}(T) = \epsilon$, with $T = \sum_{i=1}^{n-1} T_{i\to i+1}$. At all times t, only two genotypes have nonzero frequencies.

In the infinite population limit, we can write the diffusion limit of population genetics as a replicator equation

$$\frac{\partial f_i}{\partial t} = f_i(X_i - \overline{X}(t)), \quad i \in 1, \ldots, n \tag{S3.4}$$

with $\overline{X}(t)$ representing the mean fitness of the population. If we a consider a time segment $T_{i\to i+1}$ for $i \in \{1, \ldots, n-1\}$, the mean population only involves two frequencies:

$$\begin{aligned} \overline{X}(t) &= X_i f_i(t) + X_{i+1} f_{i+1}(t) \\ &= X_i f_i(t) + X_{i+1}(1 - f_i(t)), \end{aligned} \tag{S3.5}$$

so we have

$$\frac{\partial f_i}{\partial t} = -\Delta X_i f_i(t)(1 - f_i(t)), \quad i \in 1, \ldots, n \tag{S3.6}$$

bioRxiv preprint doi: https://doi.org/10.64898/2026.07.18.739367; this version posted July 20, 2026. The copyright holder for this preprint (which was not certified by peer review) is the author/funder, who has granted bioRxiv a license to display the preprint in perpetuity. It is made available under a CC-BY 4.0 International license.

where $\Delta X_i = X_{i+1} - X_i > 0$ and is defined for $i \in 1, \ldots, n$. Equation (S3.6) has an exact solution:

$$f_i(t) = \frac{1-\epsilon}{1-\epsilon+\epsilon e^{\Delta X_i t}}, \tag{S3.7}$$

where we have, without loss of generality, shifted the time so that the start of the sweep is at $t = 0$ (even if $i > 1$). By setting $f_i(T_{i\to i+1}) = \epsilon$, we can find the time interval $T_{i\to i+1}$ over which the sweep occurs.

$$\begin{aligned} f_i(T_{i\to i+1}) &= \frac{1-\epsilon}{1-\epsilon+\epsilon e^{\Delta X_i T_{i\to i+1}}} \\ \Rightarrow \quad T_{i\to i+1} &= \frac{2}{\Delta X_i}\log\left(\frac{1-\epsilon}{\epsilon}\right). \end{aligned} \tag{S3.8}$$

In the interval $T_{i\to i+1}$, the the integral of the frequency is its average (1/2, by symmetry) times the time duration

$$\int_0^{T_{i\to i+1}} f_i(t) = \int_0^{T_{i\to i+1}} f_{i+1}(t) = \frac{T_{i\to i+1}}{2} = \frac{1}{\Delta X_i}\log\left(\frac{1-\epsilon}{\epsilon}\right). \tag{S3.9}$$

The integral of the frequency squared is exactly computable (both genotypes i and $i+1$ have the same value of the integrated frequency squared, by symmetry):

$$\int_0^{T_{i\to i+1}} [f_i(t)]^2 = \int_0^{T_{i\to i+1}} [f_{i+1}(t)]^2 = \frac{2\epsilon - 1 + \log\left(\frac{1-\epsilon}{\epsilon}\right)}{\Delta X_i}. \tag{S3.10}$$

We now must compute the elements the matrix in eq. (S3.3). We first start with the general $i \in 2, \ldots, n-1$, excluding the first and last genotypes. Each average will be built from contributions from two time segments, $T_{i-1\to i}$ and $T_{i\to i+1}$, which will be the sum of two of the integrals computed above:

$$\begin{aligned} T\langle f_i(t)\rangle &= \frac{1}{\Delta X_{i-1}}\log\left(\frac{1-\epsilon}{\epsilon}\right) + \frac{1}{\Delta X_i}\log\left(\frac{1-\epsilon}{\epsilon}\right) \\ &= \left(\frac{1}{\Delta X_{i-1}} + \frac{1}{\Delta X_i}\right)\log\left(\frac{1-\epsilon}{\epsilon}\right), \end{aligned} \tag{S3.11}$$

and

$$\begin{aligned} T\left\langle f_i(t)^2\right\rangle &= \frac{2\epsilon - 1 + \log\left(\frac{1-\epsilon}{\epsilon}\right)}{\Delta X_{i-1}} + \frac{2\epsilon - 1 + \log\left(\frac{1-\epsilon}{\epsilon}\right)}{\Delta X_i} \\ &= \left(\frac{1}{\Delta X_{i-1}} + \frac{1}{\Delta X_i}\right)\left(2\epsilon - 1 + \log\left(\frac{1-\epsilon}{\epsilon}\right)\right). \end{aligned} \tag{S3.12}$$

Thus, we can get the diagonal elements of the matrix for $i \in \{2, \ldots, n-1\}$:

$$T\left(\langle f_i(t)\rangle - \left\langle f_i(t)^2\right\rangle\right) = \left(\frac{1}{\Delta X_{i-1}} + \frac{1}{\Delta X_i}\right)(1-2\epsilon). \tag{S3.13}$$

bioRxiv preprint doi: https://doi.org/10.64898/2026.07.18.739367; this version posted July 20, 2026. The copyright holder for this preprint (which was not certified by peer review) is the author/funder, who has granted bioRxiv a license to display the preprint in perpetuity. It is made available under a CC-BY 4.0 International license.

For $i \in \{1, \ldots, n-1\}$, the off-diagonal elements are also simply computable:

$$\begin{aligned} -T \langle f_i(t) f_{i+1}(t) \rangle &= -\int_0^{T_{i\to i+1}} \mathrm{d}t\, f_i(t) f_{i+1}(t) \\ &= -\int_0^{T_{i\to i+1}} \mathrm{d}t\, f_i(t)(1 - f_i(t)) \\ &= -\int_0^{T_{i\to i+1}} \mathrm{d}t\, f_i(t) + \int_0^{T_{i\to i+1}} \mathrm{d}t\, [f_i(t)]^2 \\ &= -\frac{1-2\epsilon}{\Delta X_i}. \end{aligned} \tag{S3.14}$$

The last two terms left to calculate are the diagonal elements for genotypes 1 and n:

$$\begin{aligned} T \left(\langle f_1(t) \rangle - \langle f_1(t)^2 \rangle \right) &= \int_0^{T_{1\to 2}} \mathrm{d}t\, f_i(t) - \int_0^{T_{1\to 2}} \mathrm{d}t\, [f_1(t)]^2 \\ &= \frac{1-2\epsilon}{\Delta X_1}, \end{aligned} \tag{S3.15}$$

and

$$\begin{aligned} T \left(\langle f_n(t) \rangle - \langle f_n(t)^2 \rangle \right) &= \int_0^{T_{n-1\to n}} \mathrm{d}t\, f_n(t) - \int_0^{T_{n-1\to n}} \mathrm{d}t\, [f_n(t)]^2 \\ &= \int_0^{T_{n-1\to n}} \mathrm{d}t\, f_{n-1}(t) - \int_0^{T_{n-1\to n}} \mathrm{d}t\, [f_{n-1}(t)]^2 \\ &= \frac{1-2\epsilon}{\Delta X_{n-1}}, \end{aligned} \tag{S3.16}$$

where in the second equality we have used the symmetry of the trajectories.

We now define the matrix

$$C_{ij} = \begin{cases} \dfrac{1}{\Delta X_1}, & i = j = 1 \\ \dfrac{1}{\Delta X_{i-1}} + \dfrac{1}{\Delta X_i}, & i = j \\ -\dfrac{1}{\Delta X_{i-1}}, & |i-j| = 1 \\ \dfrac{1}{\Delta X_{n-1}}, & i = j = n, \end{cases} \tag{S3.17}$$

which is a tridiagonal, symmetric matrix. Bringing out a factor of $\frac{1-2\epsilon}{T}$, we have

$$\begin{aligned} \frac{1-2\epsilon}{T} C\mathbf{X} &= \frac{\mathbf{f}(T) - \mathbf{f}(0)}{T} \\ \Rightarrow \quad \frac{1-2\epsilon}{1-\epsilon} C\mathbf{X} &= \mathbf{e}_n - \mathbf{e}_1, \end{aligned} \tag{S3.18}$$

where $\mathbf{e}_i$ is a unit vector pointing in the i-th direction. Absorbing the constant $\frac{1-2\epsilon}{1-\epsilon}$ into the fitness vector $\widetilde{\mathbf{X}} \equiv \frac{1-2\epsilon}{1-\epsilon}\mathbf{X}$, we have

$$C\widetilde{\mathbf{X}} = \mathbf{e}_n - \mathbf{e}_1. \tag{S3.19}$$

bioRxiv preprint doi: https://doi.org/10.64898/2026.07.18.739367; this version posted July 20, 2026. The copyright holder for this preprint (which was not certified by peer review) is the author/funder, who has granted bioRxiv a license to display the preprint in perpetuity. It is made available under a CC-BY 4.0 International license.

Computing the exact pseudoinverse C^+ is difficult, but we can recall that $C^+(\mathbf{e}_1 - \mathbf{e}_n)$ will be provide the minimum least squares solution $\hat{\widetilde{\mathbf{X}}}$ with the smallest ℓ_2 norm, minimizing

$$\mathcal{L}(\widetilde{\mathbf{X}}) = \frac{1}{2}\left\|C\widetilde{\mathbf{X}} - (\mathbf{e}_n - \mathbf{e}_1)\right\|_2^2, \tag{S3.20}$$

which may not have a unique solution. Using these two facts of the pseudoinverse, we will now exactly compute $C^+(\mathbf{e}_1 - \mathbf{e}_n)$.

First, we show that the original fitness vector $\mathbf{X}$ minimizes the least squares loss. First, we compute $C\mathbf{X}$:

$$\begin{aligned}
(C\mathbf{X})_1 &= \frac{X_1}{\Delta X_1} - \frac{X_2}{\Delta X_1} = \frac{-(X_2 - X_1)}{X_2 - X_1} = -1, \\
(C\mathbf{X})_i &= -\frac{X_i}{\Delta X_{i-1}} - \frac{X_{i+1}}{\Delta X_i} + X_i\left(\frac{1}{\Delta X_{i-1}} + \frac{1}{\Delta X_i}\right) = 0, \\
(C\mathbf{X})_n &= -\frac{X_{n-1}}{\Delta X_{n-1}} + \frac{X_n}{\Delta X_{n-1}} = \frac{X_n - X_{n-1}}{X_n - X_{n-1}} = 1,
\end{aligned} \tag{S3.21}$$

where $i \in \{2, \ldots, n-1\}$, so we can write

$$C\mathbf{X} = \mathbf{e}_n - \mathbf{e}_1. \tag{S3.22}$$

Thus, $\mathcal{L}(\widetilde{\mathbf{X}} = \mathbf{X}) = 0$. Since $\mathcal{L}(\widetilde{\mathbf{X}}) \geq 0$, we know that $\mathcal{L}(\widetilde{\mathbf{X}}) = 0$ minimizes the least squares loss. However, we do not know if this solution is unique, and if it not unique, then which solution would be returned by $\hat{\widetilde{\mathbf{X}}} = C^+(\mathbf{e}_1 - \mathbf{e}_n)$.

Now, we prove that the solution is not unique because C is not invertible. We show this by noting that $\mathbb{1}$, the vector of all ones, is in the kernel of C:

$$\begin{aligned}
(C\mathbb{1})_1 &= \frac{1}{\Delta X_1} - \frac{1}{\Delta X_1} = 0, \\
(C\mathbb{1})_i &= -\frac{1}{\Delta X_{i-1}} - \frac{1}{\Delta X_i} + \left(\frac{1}{\Delta X_{i-1}} + \frac{1}{\Delta X_i}\right) = 0, \\
(C\mathbb{1})_n &= -\frac{1}{\Delta X_{n-1}} + \frac{1}{\Delta X_{n-1}} = 0.
\end{aligned} \tag{S3.23}$$

so we can write

$$C\mathbb{1} = \mathbf{0}. \tag{S3.24}$$

Thus, C is rank-deficient and therefore singular. Additional least squares-minimizing solutions can thus be constructed by adding any multiple of the homogeneous solution $\mathbb{1}$ and the particular solution $\mathbf{X}$. This makes physical sense because the original replicator equations are invariant to constant shifts in the fitness.

We now must show that the space of all possible solutions has been found and that there are no other homogeneous solution. We do this by showing that $\text{rank}(C) = n - 1$. Noting that C is a symmetric tridiagonal matrix, we first define an $(n-1) \times (n-1)$ matrix B such that

$$B_{ij} \equiv C_{i+1,j}, \quad i \in \{1, \ldots, n-1\}. \tag{S3.25}$$

bioRxiv preprint doi: https://doi.org/10.64898/2026.07.18.739367; this version posted July 20, 2026. The copyright holder for this preprint (which was not certified by peer review) is the author/funder, who has granted bioRxiv a license to display the preprint in perpetuity. It is made available under a CC-BY 4.0 International license.

B is an upper triangular matrix whose diagonals are known. The determinant of an upper triangular matrix are given by the product of its diagonal elements, so we have

$$\det(B) = (-1)^{n-1} \prod_{i=1}^{n-1} \frac{1}{\Delta X_{n-1}} \neq 0. \tag{S3.26}$$

Since the determinant of B is nonzero, we know that B is full rank. Now, since B is a submatrix of C, we must have that $\text{rank}(C) \geq \text{rank}(B) = n - 1$. Since we have found a homogeneous solution to $C\widetilde{\mathbf{X}} = 0$, it immediately follows that $\text{rank}(C) \leq n - 1$. Together, this means we have $\text{rank}(C) = n - 1$.

Therefore the set of all least squares-minimizing solutions is given by a linear combination of $\mathbf{X} + \lambda\mathbb{1}$. We now must find $\hat{\tilde{\mathbf{X}}} = C^+(\mathbf{e}_1 - \mathbf{e}_n)$, which will be given by the solution $\mathbf{X} + \lambda\mathbb{1}$ with λ chosen such that $\|\mathbf{X} + \lambda\mathbb{1}\|_2^2$ is minimized. We do this by direct optimization:

$$\begin{aligned} 0 = \frac{\partial}{\partial\lambda}\|\mathbf{X} + \lambda\mathbb{1}\|_2^2 = \frac{\partial}{\partial\lambda}\left(\mathbf{X}\cdot\mathbf{X} + 2\lambda(\mathbf{X}\cdot\mathbb{1}) + \lambda^2(\mathbb{1}\cdot\mathbb{1})\right) \\ 2\lambda\sum_{i=1}^{n} X_i + 2n\lambda, \end{aligned} \tag{S3.27}$$

from which it follows that

$$\lambda = -\frac{1}{n}\sum_{i=1}^{n} X_i \equiv -\frac{\|\mathbf{X}\|_1}{n}, \tag{S3.28}$$

which is the negative of the mean of the entire fitness vector.

We now have that the exact inferred fitness vector given by eq. (S3.3)

$$\hat{\tilde{\mathbf{X}}} = C^+(\mathbf{e}_1 - \mathbf{e}_n) = \mathbf{X} - \frac{\|\mathbf{X}\|_1}{n}\mathbb{1}. \tag{S3.29}$$

Indeed, the Pearson correlation $r = 1$ since the inferred fitness vector $\hat{\tilde{\mathbf{X}}}$ and true fitness vector $\mathbf{X}$ are linearly related. The proof is complete.

S4 Derivation of negative identity matrix approximation for scaled mutation matrix for large genotype spaces

Suppose there are G_{poss} genotypes that can be observed, and G_{obs} genotypes that are actually observed. Suppose we define the mutation matrix M to be a $G_{\text{poss}} \times G_{\text{poss}}$ matrix on the genotype space of all *possible* genotypes, not necessarily just the observed ones; accordingly, $\langle \mathbf{f}(t)\rangle_t$ is a vector of length G_{poss} corresponding to the time-averaged frequency of all possible genotypes, observed and unobserved.

The mutation rate matrix has the same definition as before: $M_{gg'} = \mu_{g'\to g}$ for $g \neq g'$ and $M_{gg} = -\sum_{g'\neq g}\mu_{g\to g'}$. We had also defined a rescaled relative mutation rate matrix $\widetilde{M} = \alpha M$, with α being a scaling factor that does not have to be known *a priori*. In Equation (S3.3), the

bioRxiv preprint doi: https://doi.org/10.64898/2026.07.18.739367; this version posted July 20, 2026. The copyright holder for this preprint (which was not certified by peer review) is the author/funder, who has granted bioRxiv a license to display the preprint in perpetuity. It is made available under a CC-BY 4.0 International license.

matrix $\widetilde{M}\left\langle \mathbf{f}(t)\right\rangle_t = \alpha M\left\langle \mathbf{f}(t)\right\rangle_t$ appears in the computation. For a genotype g, we can write an element of the vector

$$\begin{aligned}(M\left\langle \mathbf{f}(t)\right\rangle_t)_g &= \underbrace{M_{gg}\left\langle f_g(t)\right\rangle_t}_{\text{outgoing mutations}} + \underbrace{\sum_{g'\neq g} M_{gg'}\left\langle f_{g'}(t)\right\rangle_t}_{\text{incoming mutations}} \\ &= \underbrace{-\sum_{g'\neq g}\mu_{g\to g'}\left\langle f_g(t)\right\rangle_t}_{\text{outgoing mutations}} + \underbrace{\sum_{g'\neq g}\mu_{g'\to g}\left\langle f_{g'}(t)\right\rangle_t}_{\text{incoming mutations}} \\ &= \underbrace{-\sum_{g'\in \mathrm{nn}(g)}\mu_{g\to g'}\left\langle f_g(t)\right\rangle_t}_{\text{outgoing mutations}} + \underbrace{\sum_{g'\in \mathrm{nn}(g)}\mu_{g'\to g}\left\langle f_{g'}(t)\right\rangle_t}_{\text{incoming mutations}},\end{aligned} \tag{S4.1}$$

where we have used $\mathrm{nn}(g)$ to denote the *set* of genotypes which are nearest neighbors of genotype g. Also, let $n_{\mathrm{poss}}(g) \equiv |\mathrm{nn}(g)|$ be the *number* of possible outgoing mutational neighbors from genotype g.

Now, suppose that $\mu_{g\to g'}$ and $\mu_{g'\to g}$ are only nonzero if genotypes g and g' are neighbors in the genotype space (e.g. accessible via a point mutation). We then work in the approximation that a genotype mutation from g to any neighboring genotype g' happens with probability $\mu_{g\to g'} \equiv \mu/n_{\mathrm{poss}}(g)$. This means that any outgoing mutation has uniform probability of going from genotype g to any of its $n_{\mathrm{poss}}(g)$ neighbors with uniform probability, and the total outgoing mutational probability from any genotype g is

$$\sum_{g'\in \mathrm{nn}(g)}\mu_{g\to g'} = \sum_{g'\in \mathrm{nn}(g)}\frac{\mu}{n_{\mathrm{poss}}(g)} = \frac{\mu}{n_{\mathrm{poss}}(g)}\sum_{g'\in \mathrm{nn}(g)} 1 = \frac{\mu}{n_{\mathrm{poss}}(g)} n_{\mathrm{poss}}(g) = \mu. \tag{S4.2}$$

Therefore, we can also write

$$\begin{aligned}-\sum_{g'\in \mathrm{nn}(g)}\frac{\mu}{n_{\mathrm{poss}}(g)}\left\langle f_g(t)\right\rangle_t &= -\frac{\mu}{n_{\mathrm{poss}}(g)}\left\langle f_g(t)\right\rangle_t\sum_{g'\in \mathrm{nn}(g)} 1 = -\frac{\mu}{n_{\mathrm{poss}}(g)}\left\langle f_g(t)\right\rangle_t n_{\mathrm{poss}}(g) \\ &= -\mu\left\langle f_g(t)\right\rangle_t.\end{aligned} \tag{S4.3}$$

For incoming mutations, we can write

$$\sum_{g'\in \mathrm{nn}(g)}\mu_{g'\to g}\left\langle f_{g'}(t)\right\rangle_t = \sum_{g'\in \mathrm{nn}(g)}\frac{\mu}{n_{\mathrm{poss}}(g')}\left\langle f_{g'}(t)\right\rangle_t. \tag{S4.4}$$

If we assume that the number of neighbors is the same for every genotype (as is the case for fixed genome lengths with fixed alphabets), then we can further write

$$\sum_{g'\in \mathrm{nn}(g)}\mu_{g'\to g}\left\langle f_{g'}(t)\right\rangle_t = \frac{\mu}{n_{\mathrm{poss}}}\sum_{g'\in \mathrm{nn}(g)}\left\langle f_{g'}(t)\right\rangle_t = \mu\mathbb{E}_{g'\in \mathrm{nn}(g),\mathrm{poss}}\left[\left\langle f_{g'}(t)\right\rangle_t\right], \tag{S4.5}$$

where $\mathbb{E}_{g'\in \mathrm{nn}(g),\mathrm{poss}}\left[\left\langle f_{g'}(t)\right\rangle_t\right]$ is average of the mean frequencies of all neighbors of genotype g. Then, the diagonal term in the mutation matrix dominates over the off-diagonals, for a

bioRxiv preprint doi: https://doi.org/10.64898/2026.07.18.739367; this version posted July 20, 2026. The copyright holder for this preprint (which was not certified by peer review) is the author/funder, who has granted bioRxiv a license to display the preprint in perpetuity. It is made available under a CC-BY 4.0 International license.

genotype g, if

$$\frac{\langle f_g(t)\rangle_t}{\mathbb{E}_{g'\in \mathrm{nn}(g),\mathrm{poss}}\left[\langle f_{g'}(t)\rangle_t\right]} \gg 1, \tag{S4.6}$$

which means the observe frequency of genotype g should greatly exceed the frequencies of its neighbors. We argue that in very large genotype spaces, the neighbor-averaged mean frequency $\mathbb{E}_{g'\in \mathrm{nn}(g),\mathrm{poss}}\left[\langle f_{g'}(t)\rangle_t\right]$ will strongly be skewed by a large number of zeros due to the vast majority of genotypes going unobserved; thus, the condition eq. (S4.6) should be valid for experimental settings like barcode experiments and microbial evolution experiments with long-read sequencing.

To strengthen the argument and provide better intepretation for eq. (S4.6), we can continue: writing $n_{\mathrm{obs}}(g)$ as the number of *observed* neighbors of genotype g, we can also rewrite the expectation $\mathbb{E}_{g'\in \mathrm{nn}(g),\mathrm{poss}}\left[\langle f_{g'}(t)\rangle_t\right]$

$$\mathbb{E}_{g'\in \mathrm{nn}(g),\mathrm{poss}}\left[\langle f_{g'}(t)\rangle_t\right] = \frac{n_{\mathrm{obs}}(g)}{n_{\mathrm{poss}}}\mathbb{E}_{g'\in \mathrm{nn}(g),\mathrm{obs}}\left[\langle f_{g'}(t)\rangle_t\right], \tag{S4.7}$$

where $\mathbb{E}_{g'\in \mathrm{nn}(g),\mathrm{obs}}\left[\langle f_{g'}(t)\rangle_t\right]$ is the expectation of average frequency of the observed neighboring genotypes of genotype g. This yields an equivalent condition

$$\frac{\langle f_g(t)\rangle_t}{\mathbb{E}_{g'\in \mathrm{nn}(g),\mathrm{obs}}\left[\langle f_{g'}(t)\rangle_t\right]} \gg \frac{n_{\mathrm{obs}}(g)}{n_{\mathrm{poss}}}. \tag{S4.8}$$

Again, the ratio of observed neighbors $n_{\mathrm{obs}}(g)/n_{\mathrm{poss}}$ is likely to be very small $n_{\mathrm{obs}}(g)/n_{\mathrm{poss}} \ll 1$, and is guaranteed to be less than or equal to 1: $n_{\mathrm{obs}}(g)/n_{\mathrm{poss}} \le 1$. Then, if genotype g is a fitness peak, then it is more likely to be selected over time and more abundant in the time series compared to its neighbors, so we would expect $\frac{\langle f_g(t)\rangle_t}{\mathbb{E}_{g'\in \mathrm{nn}(g),\mathrm{obs}}\left[\langle f_{g'}(t)\rangle_t\right]} > 1$. This, in addition to sparse observation of the vast genotype space ($n_{\mathrm{obs}}(g)/n_{\mathrm{poss}} \ll 1$), would suggest that eq. (S4.8) should hold. If genotype g has its frequency close to the average of its neighbors (for example, possibly because g is a member of a neutral set or otherwise has comparable fitness to its neighbors), then $\frac{\langle f_g(t)\rangle_t}{\mathbb{E}_{g'\in \mathrm{nn}(g),\mathrm{obs}}\left[\langle f_{g'}(t)\rangle_t\right]} \approx 1$, and eq. (S4.8) still holds because of the sparsity of the observed subset of the genotype space. Only if genotype g is rare compared to all of its observed neighbors would we have $\frac{\langle f_g(t)\rangle_t}{\mathbb{E}_{g'\in \mathrm{nn}(g),\mathrm{obs}}\left[\langle f_{g'}(t)\rangle_t\right]} < 1$. This could occur, for instance, if g occurs in a sharp fitness valley. Even then, an additional condition is required; namely, that many of genotype g's possible neighbors are observed: $\frac{n_{\mathrm{obs}}(g)}{n_{\mathrm{poss}}} \sim 1$. Only then is eq. (S4.8) likely to be violated.

Based on this interpretation, we believe that for large genotype spaces, eq. (S4.6) (and, equivalently, eq. (S4.8)) is likely to be true, which would mean that contributions to each element of $M\langle \mathbf{f}(t)\rangle_t$ from outgoing mutations will outweigh the sum of contributions from incoming mutations:

$$\underbrace{M_{gg}\langle f_g(t)\rangle_t}_{\text{outgoing mutations}} \gg \underbrace{\sum_{g'\neq g} M_{gg'}\langle f_{g'}(t)\rangle_t}_{\text{incoming mutations}}. \tag{S4.9}$$

bioRxiv preprint doi: https://doi.org/10.64898/2026.07.18.739367; this version posted July 20, 2026. The copyright holder for this preprint (which was not certified by peer review) is the author/funder, who has granted bioRxiv a license to display the preprint in perpetuity. It is made available under a CC-BY 4.0 International license.

Then, it follows that

$$\begin{aligned}
(M \langle \mathbf{f}(t) \rangle_t)_g &= \underbrace{M_{gg} \langle f_g(t) \rangle_t}_{\text{outgoing mutations}} + \underbrace{\sum_{g' \neq g} M_{gg'} \langle f_{g'}(t) \rangle_t}_{\text{incoming mutations}} \\
&\approx \underbrace{M_{gg} \langle f_g(t) \rangle_t}_{\text{outgoing mutations}} \\
&= - \langle f_g(t) \rangle_t \sum_{g' \neq g} \mu_{g \to g'} .
\end{aligned} \tag{S4.10}$$

Using $\mu_{g \to g'} = \mu / n_{\text{poss}}(g)$, this simplifies to

$$(M \langle \mathbf{f}(t) \rangle_t)_g \approx -\mu \langle f_g(t) \rangle_t \, , \tag{S4.11}$$

or

$$M \langle \mathbf{f}(t) \rangle_t \approx -\mu \mathbb{I} \langle \mathbf{f}(t) \rangle_t \, . \tag{S4.12}$$

When using the scaled mutation matrix $\widetilde{M} = \alpha M$, we can simply absorb the mutation rate μ into the positive constant α, writing

$$\widetilde{M} \langle \mathbf{f}(t) \rangle_t \approx -\mathbb{I} \langle \mathbf{f}(t) \rangle_t \tag{S4.13}$$

In the fitness estimation equation eq. (S1.5), the mutation matrix only appears in the context of acting as an operator on the average frequency vector. Therefore, we can replace $\widetilde{M} \mapsto -\mathbb{I}$ when the mutation matrix is not known or difficult to calculate, and if the genotype space is very large. In the Main Text we show that this works well for barcoded cell growth simulations, two real barcoded yeast evolution experiments, and two murine norovirus 1 serial evolution experiments which use long-read sequencing.

bioRxiv preprint doi: https://doi.org/10.64898/2026.07.18.739367; this version posted July 20, 2026. The copyright holder for this preprint (which was not certified by peer review) is the author/funder, who has granted bioRxiv a license to display the preprint in perpetuity. It is made available under a CC-BY 4.0 International license.

S5 Supplementary Figures

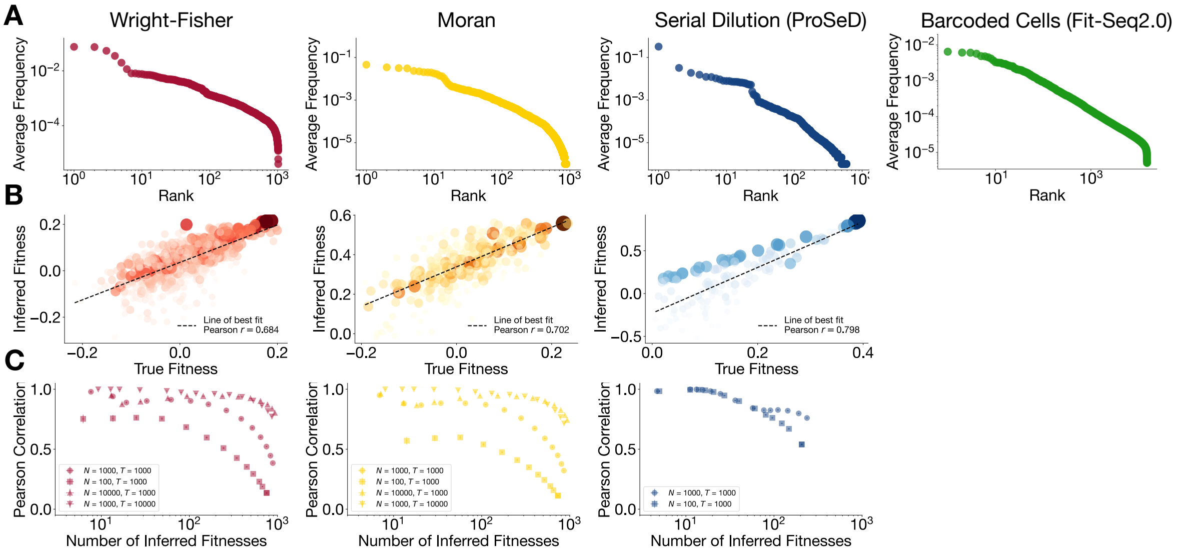

Figure S1: **Extended data for Main Text Figure 2: Fitness inference for simulated evolutionary dynamics using Main Text eq. (2).** (**A**) Average genotype frequency versus rank for Wright-Fisher, Moran, ProSeD, and Fit-Seq2.0 Growth Simulations, demonstrating $\gtrsim 4$ orders of magnitude variation in genotype average frequency. (**B**) Comparison between ground truth fitnesses and fitnesses inferred using Main Text eq. (2) on example trajectories for each simulation type, evaluating on genotypes whose average frequency was $\langle f(t) \rangle_t \geq 10^{-4}$. Main Text eq. (2) demonstrates faithful reconstruction of the observed fitnesses even for rare genotypes. (**C**) Pearson correlation between ground-truth fitnesses and fitnesses inferred using Main Text eq. (2), as a function of number of inferred fitnesses, which is set by the average frequency cutoff. Multiple population sizes and/or simulation lengths are shown. Error bars are standard errors.

bioRxiv preprint doi: https://doi.org/10.64898/2026.07.18.739367; this version posted July 20, 2026. The copyright holder for this preprint (which was not certified by peer review) is the author/funder, who has granted bioRxiv a license to display the preprint in perpetuity. It is made available under a CC-BY 4.0 International license.

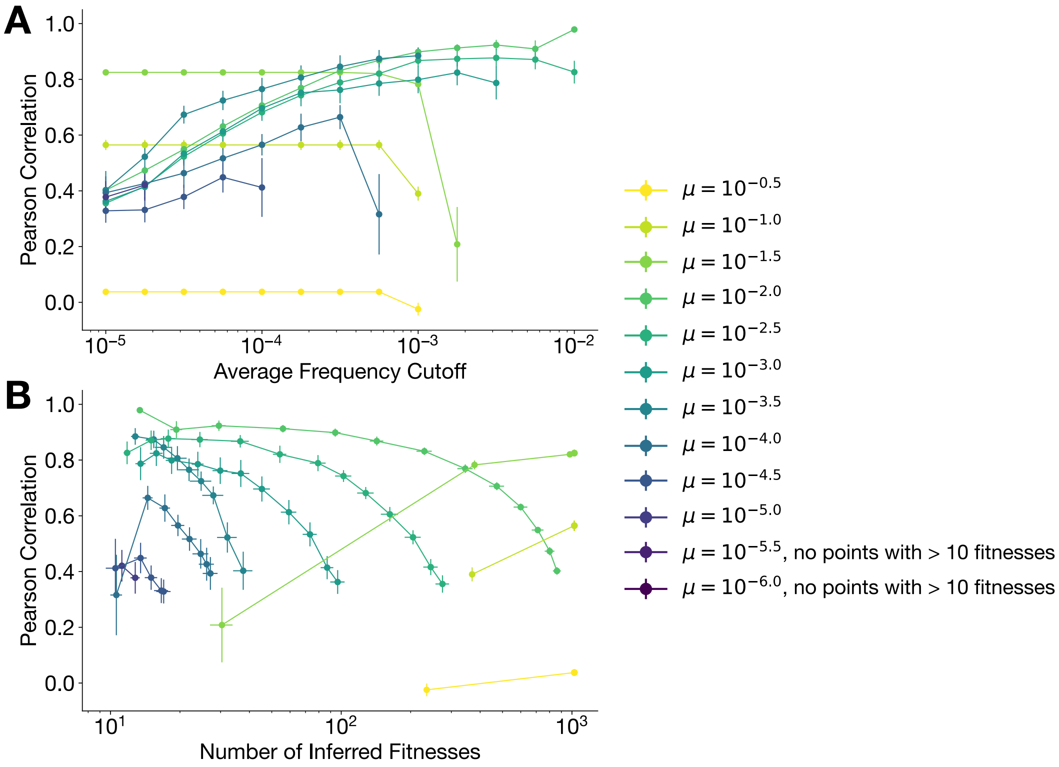

Figure S2: **Pearson correlation of recovered and true fitness landscapes for varied mutation probabilities (per-site, per-individual, per generation) in Wright-Fisher simulations.** (**A**) Pearson correlation between fitnesses inferred using Main Text eq. (2) and literature-reported fitnesses, as a function of number of inferred fitnesses, as a function of average frequency cutoff. (**B**) Pearson correlation between fitnesses inferred using Main Text eq. (2) and literature-reported fitnesses, as a function of number of inferred fitnesses, as a function of number of inferred fitnesses, which is set by the average frequency cutoff. Curves are plotted for simulations with different mutation probabilities; points are averaged over 10 trials. All curves are plotted for simulations with different mutation probabilities; points are averaged over 10 trials; error bars are standard errors.

bioRxiv preprint doi: https://doi.org/10.64898/2026.07.18.739367; this version posted July 20, 2026. The copyright holder for this preprint (which was not certified by peer review) is the author/funder, who has granted bioRxiv a license to display the preprint in perpetuity. It is made available under a CC-BY 4.0 International license.

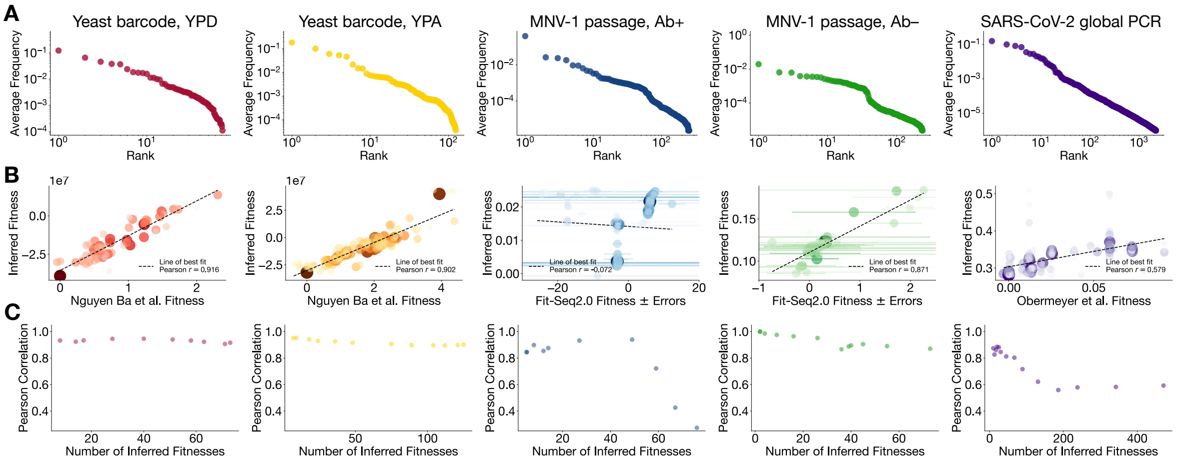

Figure S3: **Extended data for Main Text Figure 3: Fitness inference for empirical evolutionary dynamics using Main Text eq. (2).** (**A**) Average genotype frequency versus rank each empirical time series, demonstrating $\gtrsim 4$ orders of magnitude variation in genotype average frequency. (**B**) Comparison between fitnesses inferred using Main Text eq. (2) and fitnesses inferred by other methods in the literature; we included genotypes whose average frequency was $\langle f(t) \rangle_t \geq 10^{-4.5}$. Main Text eq. (2) demonstrates reconstruction of the observed fitnesses comparable to those found by other methods. (**C**) Pearson correlation between fitnesses inferred using Main Text eq. (2) and literature-reported fitnesses, as a function of number of inferred fitnesses, which is set by the average frequency cutoff.
